# Harnessing Real-Time UV Imaging and Convolutional Neural Networks (CNNs): Unlocking New Opportunities for Empirical In Vitro–In Vivo Relationship Modelling

**DOI:** 10.3390/pharmaceutics17060728

**Published:** 2025-05-31

**Authors:** Maciej Stróżyk, Adam Pacławski, Aleksander Mendyk

**Affiliations:** Department of Pharmaceutical Technology and Biopharmaceutics, Jagiellonian University Medical College, 30-688 Kraków, Poland; maciej.strozyk-guest@uj.edu.pl (M.S.); aleksander.mendyk@uj.edu.pl (A.M.)

**Keywords:** in vitro in vivo relationship, artificial intelligence (AI), surface dissolution UV imaging, SDi2, convolutional neural networks (CNN)

## Abstract

**Background:** This study delves into the potential use of real-time UV imaging of the dissolution process combined with convolutional neural networks (CNNs) to develop multidimensional models representing the relation between in vitro and in vivo performance of drugs. **Method:** We utilised the capabilities of the SDi2 apparatus (Pion) to capture multidimensional dissolution data for two distinct Glucophage tablets: immediate-release 500 mg tablets and extended-release 750 mg tablets. The dissolution process was studied in various media, including a compendial pH 1.2 HCl solution, reverse osmosis water, and pH 6.8 phosphate buffer. **Result:** Moreover, results were captured at different wavelengths (255 nm and 520 nm) to provide a comprehensive view of the process. Our investigation focuses on two primary approaches: (1) analysing numerical data extracted from SDi2 images via a surface characterisation tool, using traditional machine learning techniques, including Scikit-learn, Tensorflow, and AutoML, and (2) utilising raw SDi2 images to train CNNs for direct prediction of in vivo metformin plasma concentrations. **Conclusions:** This dual approach allows us to assess the impact of data extraction on model performance and explore the potential of CNNs to capture complex dissolution patterns directly from images, potentially revealing hidden information not captured by traditional numerical data extraction methods.

## 1. Introduction

Dissolution testing stands as a cornerstone methodology in the drug development process, serving as a bridge between drug formulation and therapeutic outcomes [1]. These simple but insightful tests quantify the rate and extent of drug release under controlled conditions, providing researchers with fundamental insights into dosage form behaviour in a dynamic environment similar to that observed after drug administration to a patient [2]. However, the pharmaceutical industry has long grappled with a fundamental challenge: the significant disparity between in vitro dissolution measurements and actual in vivo drug performance [3].

Predicting drug behaviour in the human body remains one of the most complex challenges in pharmaceutical development, especially at the dosage form level. Traditional dissolution testing methods frequently fail to accurately represent the intricate biological environment of the gastrointestinal tract, leading to significant discrepancies between laboratory measurements and actual drug performance. The primary obstacle lies in the extraordinary complexity of the digestive system, characterised by dynamically changing pH levels, fluid movements, and enzymatic activities [4]. Approximately 40% of new chemical entities demonstrate substantial challenges related to solubility and absorption, resulting in unpredictable pharmacological profiles [5]. Key challenges include highly variable pH conditions across different gastrointestinal segments, complex interactions between food components and drug molecules, diverse digestive and metabolic processes and significant inter-individual physiological variations. Traditional testing methodologies cannot adequately capture these intricate interactions, resulting in substantial divergences between laboratory findings and actual drug behaviour within the human body. The pharmaceutical industry increasingly recognises the need for more sophisticated, dynamic testing approaches that can more accurately simulate the complex in vivo environment.

One interesting solution that extends the possibility of multidimensional visualization of the dissolution process is UV real-time spectroscopy, particularly through UV dissolution imaging in a surface dissolution imaging device. By measuring the absorbance of ultraviolet light, this technique provides continuous, real-time monitoring of drug release during dissolution processes as well as macroscopic changes in dosage form like erosion, swelling, and gel layer formation. This ensures that dynamic changes, such as precipitation and phase transitions, are captured accurately [6]. This non-invasive method eliminates the need for sample extraction, thus preserving the integrity of the dissolution environment. Furthermore, UV spectroscopy’s ability to capture both temporal and spatial data enhances our understanding of drug release mechanisms across various formulations, including tablets, capsules, and hydrogels, making it invaluable for early-stage and late-stage formulation studies [7].

UV dissolution imaging produces a substantial volume of data, requiring proper analytical methods to ensure its effective use. In this context, emerging technologies such as artificial intelligence and machine learning (AI/ML) have proven highly valuable. These technologies excel in constructing predictive models capable of processing large datasets and tackling complex, nonlinear problems. Particularly noteworthy in this field are large neural networks, especially convolutional neural networks (CNNs), which specialise in image and pattern recognition tasks [8]. Deep learning, a subset of artificial intelligence, has emerged as a transformative force in pharmaceutical science, particularly in the analysis of complex, multidimensional datasets such as UV dissolution images. These images, which capture the dynamic process of a drug dissolving and releasing its active ingredients, provide critical insights into the dissolution, which has an impact on drug pharmacokinetic patterns, including time-dependent drug concentration in plasma. These are key factors in optimising drug formulations to ensure their efficacy, safety, and consistency. Traditional methods of analysing such images, often reliant on manual interpretation or basic computational models, may struggle to discern the intricate patterns and relationships embedded within the data. In contrast, deep learning techniques, notably Convolutional Neural Networks (CNNs) and Deep Neural Networks (DNNs), offer advanced, data-driven solutions that excel at extracting meaningful information from these complex datasets. By leveraging their ability to process large volumes of visual and relational data, these tools enable researchers to assess drug behaviour with unprecedented precision and efficiency, streamlining the formulation process [9].

CNNs are particularly well-suited for analysing UV dissolution images due to their specialised architecture, which is designed to process and interpret visual data. These networks consist of multiple layers, including convolutional layers that detect features like edges and textures, pooling layers that reduce spatial dimensions while preserving key information, and fully connected layers that integrate these features into a cohesive analysis. This structure allows CNNs to automatically learn and identify spatial hierarchies of features, from simple shapes to complex patterns indicative of drug release behaviours, without requiring extensive manual feature engineering. For instance, in a study by Galata et al. [8], CNNs were employed to predict tablet dissolution profiles using chemical maps derived from fast Raman imaging—a technique closely aligned with UV dissolution imaging. The study demonstrated that CNNs outperformed traditional modelling approaches, offering superior accuracy in predicting how tablets dissolve over time. This enhanced capability underscores the potential of CNNs to revolutionise the interpretation of dissolution data, providing pharmaceutical scientists with a robust tool to refine drug delivery systems.

The performance of CNNs in image-based applications is strongly influenced by both network architecture and parameter initialization. These elements play a critical role in determining how effectively a CNN can extract patterns from data and generalize to unseen examples, as poor choices in either can lead to issues like overfitting or failure to converge during training.

While CNNs shine in image-based tasks, Deep Neural Networks (DNNs) bring a complementary strength by modelling complex, nonlinear relationships within diverse datasets. DNNs feature multiple hidden layers that progressively learn abstract representations of the input data, making them adept at handling tasks where variables exhibit intricate interdependencies. In pharmaceutical science, this is invaluable for unravelling the multifaceted interactions among drug formulations, excipients (inactive ingredients), and manufacturing process parameters—all of which influence dissolution and absorption outcomes. Borjigin et al. [10] demonstrated the capability of using deep learning convolutional neural networks to analyse X-ray computed microtomography (XRCT) images of mini-tablets, enabling the prediction of dissolution performance based on physical parameters like micro-crack volume and enteric coat thickness, and revealing how these features impact drug release. This suggests that DNNs could similarly enhance UV dissolution image analysis by capturing the complex factors that govern drug behaviour, offering a deeper understanding that informs formulation optimization.

The SDi2 apparatus from Pion Inc. (East Sussex, UK) is an advanced UV imaging system designed for real-time analysis of the drug dissolution process. It features dual-wavelength UV absorbance imaging, operation between pH 1.0–10.0 and 5–40 °C, allowing up to 2 different media to be used per experiment. The size of the dosage form that can be tested by the device is limited by the size of the hole in the dissolution chamber to 12 mm in height and 24 mm in width, whereas the total observation area is 28 mm × 24 mm (height × width). Additionally, the SDi2 may not be the optimal tool for regular quality assurance dissolution testing. However, it is a promising tool for dosage form development, including In vitro In vivo Correlation/In vitro In vivo Relationship (IVIVC/IVIVR) applications, as the information gathered through surface imaging may be used to develop such models. It may also be used for optimising the formulation at the development stage of dosage forms. Allowing for real-time observation of drug dissolution in the vicinity of the formulation, polymer swelling, drug precipitation/recrystallisation [11], as well as observing the impact of super disintegrants or other excipients on drug dissolution [12]. Within this study, we highlight the potential application of the SDi2 apparatus in innovative image analysis using machine learning, a highly novel and emerging field.

Amid the growing interest in AI-based solutions, we propose that the imaging capabilities of SDi2 can be harnessed to develop models for predicting in vivo metformin blood concentrations. The algorithm utilises convolutional neural networks (CNNs), which analyse entire images from the dissolution process and autonomously determine the most significant features for accurate predictions of in vivo plasma drug concentrations, eliminating the need for human intervention in regard to image processing. A similar methodology is already demonstrating its utility in biological research [13]. A notable case involves a CNN trained on H and E-stained ventricular tissue images, achieving 99% sensitivity and 94% specificity in detecting heart failure—outperforming two expert pathologists [14]. These examples underscore the practical relevance of AI-driven image analysis, aligning with the potential of SDi2 in predictive modelling. In addition, the work previously mentioned by Galata et al., within the pharmaceutical technology field, CNN training on images made through optical coherence tomography of pharmaceutical coatings is used to evaluate the coating layer [15]. This implies that our research on such applications is innovative, as this area has not yet been thoroughly explored by research scientists.

Within this study, we aim to evaluate whether empirical IVIVR models can be developed by integrating surface dissolution imaging techniques (SDi2 apparatus) with convolutional neural network (CNN) architectures. We explored various network architectures and data representations to determine the feasibility and predictive performance of this approach. We also highlight the advantage of using CNNs, which are capable of processing the whole dissolution image, limiting the need for data extraction from the images prior to model training. We would like to highlight that the models developed within this study interpret dissolution data and make predictions on the in vivo response, and they do not themselves explain the process behind the in vivo response, which is a limitation that is important to note. However, the successful prediction of in vivo drug plasma concentrations via CNN modelling of dissolution image data is a very novel accomplishment on its own, serving as a proof of concept for future research and applications.

## 2. Materials and Methods

### 2.1. Calibration Curves of Pure Metformin

In this study, metformin hydrochloride was purchased from Pol-Aura, and Glucophage IR 500, IR 850, and XR 750, products of Merck Company, were acquired from a pharmacy in Poland. To establish calibration curves for metformin, approximately 25 mg of pure metformin hydrochloride was accurately weighed using a calibrated scale (Mettler Toledo MS105DU: specifications of the device: minimum weight 3 mg, maximum weight 120 g, readability 0.1 mg) and dissolved in 50 mL of solvent within a volumetric flask. Three different solvents were used: reverse osmosis water, 0.1 M HCl solution (pH = 1.2), and phosphate buffer (pH = 6.8). From this stock solution, two series of dilutions were prepared: a high-concentration series ranging from 0.015 mg/mL to 1.0 mg/mL and a low-concentration series ranging from 2.0 µg/mL to 14.0 µg/mL. Each concentration was analysed using a UV spectrophotometer, measuring absorbance across the 200–400 nm wavelength range, and the peak absorbance values were recorded. These values were then used to construct calibration curves, covering both concentration ranges for each solvent.

### 2.2. Dissolution Test of Glucophage Tablets

Three forms of Glucophage tablets—500 mg Immediate-Release (IR), 850 mg IR, and 750 mg Extended-Release (XR)—were analysed using the SDi2 surface UV imaging apparatus to study their dissolution behaviour, and the 850 mg form was analysed to later serve as a validation data set for the CNN modelling described in Section 2.7. Custom holders were designed to ensure the tablets remained properly positioned during the dissolution process: a metal wire cage holder for the 500 mg and 850 mg IR tablets and a 3D-printed cage holder for the slim, elongated 750 mg XR tablet. The tablets were tested in three different media: a pH 1.2 buffer simulating stomach conditions, a pH 6.8 buffer simulating the gastrointestinal tract, and reverse osmosis water, and captured at two different wavelengths, 255 nm and 520 nm, resulting in six different dissolution video captures per tablet. These media were prepared according to Pharmacopeial standards, with pH levels verified using a Mettler Toledo S210 pH meter. The dissolution was conducted in a closed-loop system, where the media circulated continuously. For the 500 mg and 850 mg IR tablets, the dissolution process was run for 1 h and 15 min, as this is the time by which the tablets in water and pH 6.8 buffer underwent near-complete dissolution. For the 500 mg IR tablet, manual sampling was performed at 5, 10, 15, 30, 45, 60, and 75 min to collect samples, which were then analysed with a UV spectrophotometer to measure absorbance and calculate the metformin concentration at each time point. Similarly, for the 750 mg XR tablet, the dissolution process was monitored over a longer period of approximately 12 h. Automatic sampling was conducted using the Vision^®^ AutoPlusTM MaximizerTM apparatus at time points of 5, 10, 15, 30, 45, 60, 120, 180, 240, 300, 360, 420, 480, 540, 600, 660, and 720 min. The collected samples were analysed with a spectrophotometer to determine the metformin concentration over time. Manual sampling was not carried out for the 850 mg IR tablet, as the data obtained during its dissolution only served as a validation data set for CNN modelling described in Section 2.7.

### 2.3. Surface Dissolution Imaging

The SDi2 apparatus utilised in this study and developed by Pion Inc. is a sophisticated scientific instrument designed for dissolution testing integrated with live surface imaging, offering advanced capabilities for pharmaceutical research. It is equipped with a 4.2-megapixel ActiPix™ detector, which captures high-resolution images of the tablet surface in both visible light and the UV spectrum. The detector has a camera frame rate of 1 frame per second per wavelength, a camera pixel size (resolution) of 13.75 μm, and is capable of capturing at different ultraviolet wavelengths: 255, 280, 300, and 320 nm ± 5 nm and at a visible wavelength of: 520 nm ± 5 nm, with the dual-wavelength capture capability. In this study, the dissolution process was captured at 255 nm and 520 nm. This dual imaging functionality enables researchers to observe the dissolution process in real-time, providing a detailed visual record of surface changes while simultaneously allowing for quantitative analysis. The SDi2 offers significant flexibility in its testing methods. It supports the use of various dissolution media and allows for the modification of flow rates through its flow cell. This adaptability enables researchers to simulate a range of physiological conditions, tailoring experiments to reflect environments like the stomach or intestines. Furthermore, the apparatus offers a closed- and open-loop media circulation system. In this study, the SDi2 is employed to investigate the dissolution behaviour of Glucophage tablets. It provides insights into the drug release process and changes in tablets under simulated physiological conditions. This detailed data is invaluable for understanding how different formulations release metformin.

### 2.4. Image Retrieval and Processing

**Figure 1:**
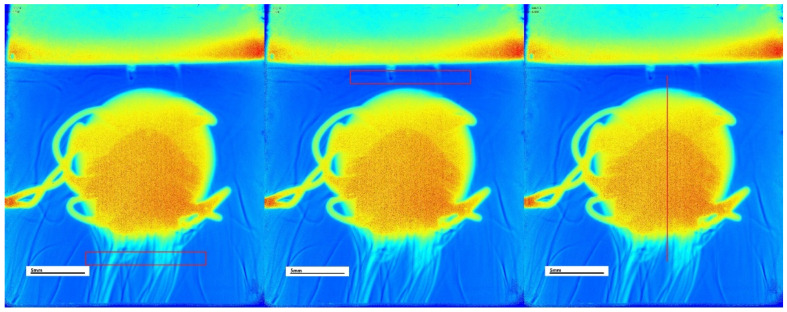
An example of the ROI selections (in red) on a single frame of the IR 500 mg tablet in pH 1.2 HCl measured at 255 nm. The first image shows the bottom rectangle ROI, followed by an image showing the top rectangle ROI, followed by the final image showing third ROI, which is the line down the height of the tablet. ROI—regions of interest. The scale is visible in the white rectangle, the black line representing 5 mm. The images utilize a jet colour scale where blue indicates the lowest absorption and red indicates the highest absorption at 255 nm. The SurfCharJ plugin version 1q [18] for ImageJ was applied to characterise ROIs by calculating local roughness, gradient analysis as well as the Kurtosis of the assessed profile, amongst other surface parameters. The final image processing procedure consisted of the manual selection and measurement of the tablet area in each frame. This involved the selection of the tablet area in all frames and the use of the built-in ImageJ measurement/analysis tool. This had to be conducted manually, as a separate tablet tracking tool/model would have to be developed to correctly select the tablet area in each frame.

The complementary software of the SDi2 apparatus developed by Pion Inc. was used to export jet-coloured videos showcasing the dissolution process. The frames were recorded every 10 s of the dissolution process and exported without further compression to VMW format. In this study, further analysis of the video data was conducted using the Python (v. 3.11) programming language and the OpenCV library [16], optimised for image and video processing. OpenCV was employed to crop the videos, isolating the region of the dissolution chamber, and it facilitated the extraction of specific frames, including the first frame in which the tablet was completely submerged and frames corresponding to predetermined sampling timepoints. The exported frames were then processed with Fiji (ImageJ version 1.54d) software [17]. The regions of interest (ROI) included a rectangular area above the tablet and below the tablet, as well as a 1-pixel wide line cutting through the tablet in the centre vertically. The areas above and below the tablet were used to characterise the area around the tablet while it was dissolving, and the whole line was used to characterise the region and size of the tablet itself while it was dissolving. The regions of interest can be seen in Figure 1.

All the different parameters calculated by the SurfCharJ plugin for each of the ROI represented different features used by the machine learning algorithm. The most important features of the best numerical model are later shown in Section 3. The first part of the feature name represents the media in which the dissolution process took place: 1_2, meaning pH 1.2 buffer; Wa, meaning Water; and 6_8, meaning pH 6.8 buffer. The second part of the feature name represents the wavelength from which that particular frame was retrieved, 255 nm and 520 nm; the last parts of the feature name are either the ROI top rectangle/line or blank, being the bottom rectangle or the parameter which was calculated. The different parameters measured by the SurfCharJ plugin are: “Ra” being the arithmetic mean deviation, “Rq” being the root mean square deviation, “Kurt” being the Kurtosis, “Rsk” being the skewness, “Min” being the minimum values in the assessment, and “StdDev” being the standard deviation measurements. An example of a feature name would be: “1_2 520top Ra”, which corresponds to the arithmetic mean deviation of the top rectangle ROI, measured at 520 nm wavelength in pH 1.2 buffer.

### 2.5. In Vivo Data Retrieval

In vivo, data represented as blood concentration over time was retrieved from three separate bioequivalence studies. In vivo plasma profiles for the 500 mg IR formulation were retrieved from the “Chinese- and French-Manufactured Immediate-Release Glucophage^®^ Bioequivalence: A Randomised, Open-Label, Crossover Study” [19]. In the case of the 750 mg XR formulation, in vivo data was retrieved from the “Bioequivalence Study of Metformin HCl XR Caplet Formulations in Healthy Indonesian Volunteers” [20]. For the validation data set of the 850 mg IR formulation, data was extracted from the “Bioequivalence Study of Two Metformin Formulations” study [21]. The time span reported in this 850 mg study only enabled the extraction of an in vivo plasma-concentration curve from 0 to 870 min, while the previously mentioned studies contained concentration-time profiles up to 24 h after administration. The data was specifically retrieved from fasted-state concentration-time profiles provided in the bioequivalence studies, with the use of Engauge Digitizer. The data was then fed into an interpolation function from the SciPy package [22] called Cubicspline.

### 2.6. Manual Numerical Modelling

In this study, we collected in vitro data, including dissolution profiles and video footage, from a 500 mg immediate-release (IR) Glucophage formulation and a 750 mg extended-release (XR) Glucophage formulation (only video footage was obtained for the 850 mg IR formulation to only serve as a validation data set for the best model developed through CNN modelling). The formulations were tested using the SDi2 apparatus across various media conditions. By processing the video footage as previously described in Section 2.4, we generated up to 443 parameters for each sampling timepoint throughout the dissolution process. These features were then used as inputs for a multidimensional in vitro in vivo relationship (IVIVR) model to predict specific points in the in vivo concentration profile of Glucophage. Given the use of multiple dissolution media and a broad set of formulation and sample parameters, the models developed in this study are more accurately classified as multidimensional IVIVR models rather than traditional IVIVC. While IVIVC aims to establish a predictive, often regulatory-accepted mathematical relationship, typically under assumptions like point-to-point correspondence between in vitro dissolution and in vivo absorption, IVIVR allows for more flexible, descriptive modelling of complex patterns without requiring a direct, predefined correlation. In this context, such high-dimensional modelling, as presented in our study, is more appropriate for IVIVR, as it captures nonlinear and high –dimensional interactions between the entire dissolution process and pharmacokinetic outcomes. Such relationships exceed the scope of conventional IVIVC frameworks and require advanced machine learning methods to model them effectively, as demonstrated in the results of this study. As previously mentioned, the models developed by these means are purely empirical, not mechanistic.

In this study, Python scripts were crafted to execute various regression analyses using the Scikit-Learn [23] and TensorFlow [24] libraries, initially developing eight distinct models with the following learning algorithms: Linear Regression, LASSO, Ridge, ElasticNet, Random Forest Regression, Support Vector Regression, Multi-Layer Perceptron, and Deep Neural Networks. Subsequently, feature engineering enhanced the process by generating an expanded dataset with polynomial features, followed by the removal of highly correlated features to refine the dataset, after which the same eight algorithms were applied to construct new models. All models were trained–tested using 10 –fold cross-validation coupled with GridSearch. All algorithms just described were trained on the numerical data generated as described in the methodology Section 2.4, from all six different dissolution video captures as described in Section 2.2. Additionally, for model comparison, the refined dataset was processed through H2O’s AutoML [25] with default settings and K-fold cross-validation, retaining eight of the top-performing models, with the best one highlighted in the results alongside its feature selection outcomes. This top model underwent SHAP (SHapley Additive exPlanations) [26] analysis, and the findings were compared to AutoML’s feature selection results, providing a comprehensive evaluation of the modelling process from manual development to automated optimisation.

### 2.7. Convolutional Neural Networks (CNN)

**Figure 2:**
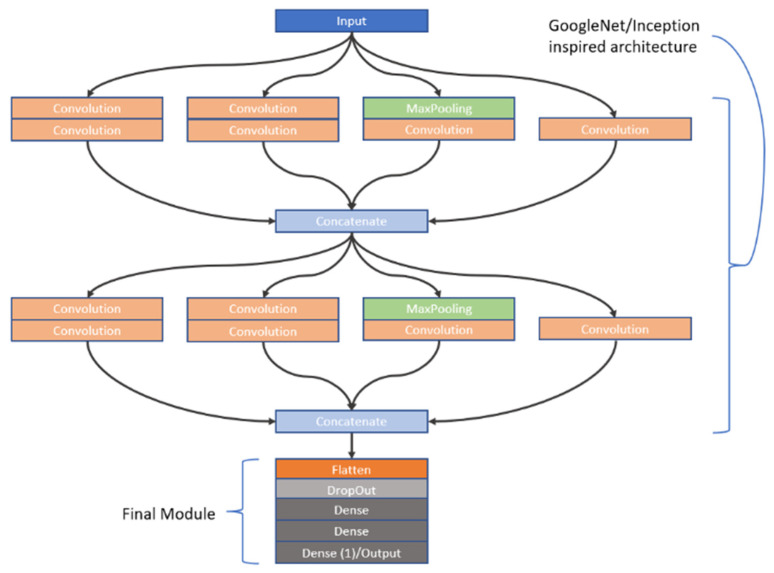
A graphical representation of the GoogleNet-inspired network. Two inception v1 modules are highlighted, which are then followed by the final module.

**Figure 3:**
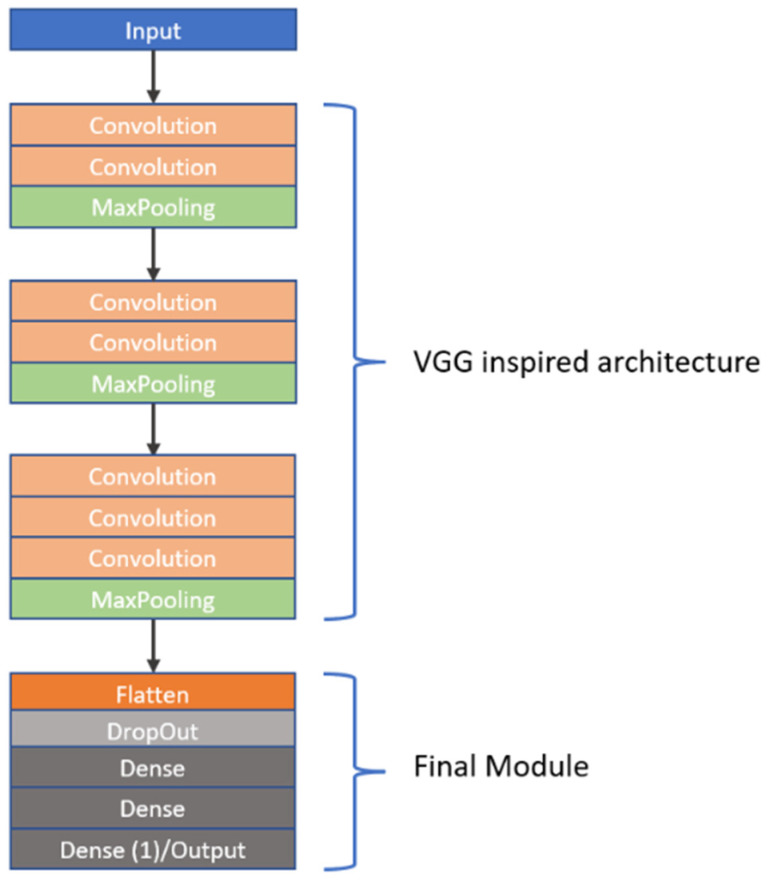
A graphical representation of the VGG-inspired network. The linear structure composed of convolutional and max-pooling layers is taken from the VGG network, followed by the final module.

**Figure 4:**
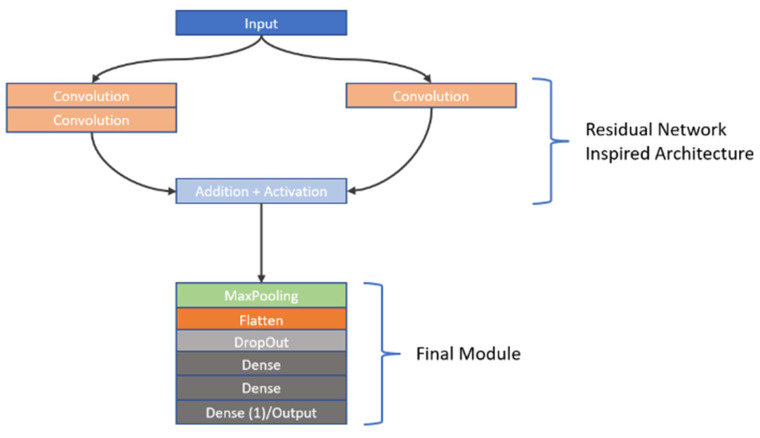
A graphical representation of the ResNet-inspired network. It consists of just 1 residual learning building block, followed by the final module.

As previously stated, CNNs are highly sensitive to their architectural design. In this study, we investigated the learning capabilities of six distinct CNN frameworks by training them on a unique dataset of image stacks derived from video frames recorded during dissolution testing of Metformin, a widely used diabetes medication, as part of our first approach to CNN modelling. Each image stack was carefully constructed to include six frames captured at the same timepoint, but these frames differed in wavelength (e.g., across the visible spectrum) and dissolution media (e.g., varying pH levels), offering a multidimensional representation of the dissolution process. This approach was chosen to reflect the dynamic chemical and physical changes occurring during dissolution, providing the CNNs with rich input data to analyse. The stacks were paired with corresponding in vivo Metformin plasma concentrations, which were interpolated from experimental pharmacokinetic data collected from human subjects, serving as the ground truth for the CNNs to predict. The six architectures we explored were split into two groups: three custom-designed models, which involved convolutions, max pooling operations, dropouts as well as batch normalization and three adapted from well-known frameworks. The custom models were built from scratch to suit the specific needs of this task, incorporating foundational CNN components. These included convolutional layers to extract spatial features like edges or textures from the dissolution images, max pooling layers to reduce computational complexity by downsampling the feature maps, dropout layers to randomly disable neurons during training and thus prevent overfitting, and batch normalisation to normalize the inputs to each layer, enhancing training stability and speed. In contrast, the second group of architectures was inspired by established models: Inception [27], VGG [28], and ResNet [29]. The Inception-inspired design leveraged multi-scale convolutions, applying filters of different sizes in parallel to capture features at varying levels of granularity, which is particularly useful for complex image data. The VGG-inspired model relied on a deep stack of sequential convolutional layers with small filters, promoting hierarchical feature learning from low-level details to high-level abstractions. Meanwhile, the ResNet-inspired architecture incorporated residual blocks—shortcuts that allow the network to skip layers—helping to address the vanishing gradient problem that often hampers very deep networks, thus enabling effective training even with increased depth. All six architectures employed the ReLU (Rectified Linear Unit) activation function after their convolutional layers to introduce nonlinearity, allowing the networks to model complex relationships within the data. However, since the original Inception, VGG, and ResNet architectures were designed for classification tasks (e.g., assigning discrete labels like “cat” or “dog” to images), they required modification for our regression task, which aimed to predict continuous Metformin concentration values. To achieve this, we appended a custom regression module to each inspired architecture, replacing their typical SoftMax or fully connected classification layers with a single output layer featuring a linear activation function. This linear output was essential for generating precise, numerical predictions of Metformin levels, aligning the models with the study’s goal of linking dissolution imagery to pharmacokinetic outcomes. To provide further insight into these adaptations, the detailed structures of the inspired architectures—Inception, VGG, and ResNet—are visually documented in Figure 2, Figure 3, and Figure 4, respectively. These figures illustrate the specific layer configurations, filter sizes, and connectivity patterns tailored for this research, offering a clear reference for understanding how each model was customized. By combining custom and inspired designs, this study not only tested the flexibility of CNNs in a novel application but also highlighted the importance of thoughtful architectural adjustments when transitioning from traditional classification to regression-based predictive modelling in pharmaceutical research.

For our first approach to CNN modelling, the frames were divided into stacks composed of six different frames; the frames in the stack shared the same time point, differing only by the wavelength and media used in each frame. This stack was then associated with an in vivo Metformin plasma concentration from the interpolated in vivo data and fed into the learning algorithm/CNN architecture.

All models were meticulously trained using K-fold cross-validation with 10 folds. This approach ensures a robust evaluation of model performance, with detailed results presented in Section 3.3.

In a novel twist on data structuring, in our second approach to CNN modelling, we departed from conventional timepoint-based stacking. Instead, we gathered all frames for each formulation—spanning various wavelengths and dissolution media—into unified stacks. These stacks were then manually enriched with an additional layer encoding the corresponding timepoint, creating a comprehensive and multidimensional input. Each stack was paired with an interpolated in vivo Metformin plasma concentration, serving as the predictive output. A total of 106 stacks were constructed this way, with 53 stacks per formulation. The temporal framework was carefully curated: the first 7 timepoints captured rapid dynamics at 0, 5, 10, 15, 30, 45, and 60 min, while the subsequent 46 timepoints were spaced 30 min apart, extending up to 1440 min (24 h). This structured progression is visually depicted in Table 1, offering a clear snapshot of the data matrix. As the dissolution process for the IR formulation was run only for 75 min and to keep data structure consistent for IR and ER formulations, the empty frames were populated in data structures, so the model makes predictions based on the multidimensional representation of the dissolution process up to 75th minute. In the case of this voxel time, information was changing, resulting in information lacking data.

To rigorously assess the models’ generalization ability, the final six records from in vivo profiles and relevant in vitro descriptions of each formulation were excluded from the training set. These withheld records were reserved as a test/extrapolation set, enabling us to evaluate the models’ performance on unseen data—a critical measure of their stability in terms of generated predictions.

The test/extrapolation set was left out on purpose to allow the algorithm to learn the dissolution process for both formulations up to a specific timepoint and later use the model to predict the 6 unseen records, creating an extrapolation scenario.

#### Validation

In addition to the described extrapolation approach, we also dedicated the whole dissolution data set of the 850 mg IR formulation for validation purposes. The CNN models described in this section were only trained on 500 mg IR and 750 mg XR dissolution data. However, the best-performing model was used to predict in vivo Metformin concentrations based on unseen dissolution data from the 850 mg IR formulation. The prediction was validated against 850 mg IR in vivo data extracted from bioequivalence studies according to Section 2.5.

## 3. Results

### 3.1. Calibration Curves

The calibration curves for pure Metformin were developed to relate concentration to absorbance across different media—water, pH 1.2, and pH 6.8—enabling accurate quantification at both high and low concentrations. For low concentrations, these curves were consistently measured at 230 nm, the wavelength corresponding to the peak absorption point. In contrast, high-concentration curves required adjustments due to excessive absorbance at 230 nm, which could lead to saturation; thus, they were obtained at 258 nm for water, 241 nm for pH 1.2, and 254 nm for pH 6.8. The coefficient of determination varied from 0.978 to 1.00 for high concentrations. For low concentrations, the R^2^ values varied from 0.999 to 1.00.

### 3.2. Numerical Data-Based Modelling

Results obtained via numerical data modelling described in Section 2.6 of the methodology are presented in Table 2 below. The models developed were assessed based on their R^2^ and RMSE values. The H2O model was the most accurate model developed, with the RMSE being the lowest at 82 and R^2^ being the highest at 0.95.

**Figure 5:**
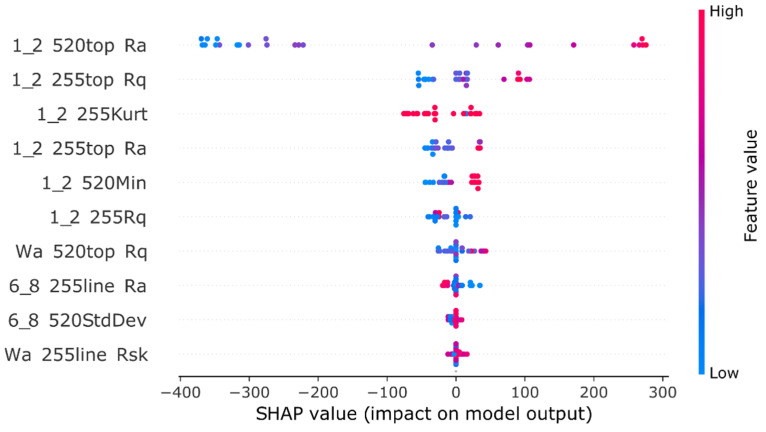
SHAP analysis of the H2O AutoML generated model, displaying the SHAP value or impact on model output of the 10 most impactful features/parameters. The first part of the feature name represents the media in which the dissolution process took place (1_2 meaning pH 1.2 buffer, Wa meaning Water and 6_8 meaning pH 6.8 buffer), the second part of the name represents the wavelength from which that particular frame was retrieved (255 and 520), the last parts of the feature name, are either the ROI (top rectangle/line or blank being the bottom rectangle) or the parameter which was calculated, as described in the methodology Section 2.4. In this case, the feature values are represented by colour, high feature value being a shade of red, and a low feature value being a shade of blue. Ra: Arithmetic mean deviation; Rq: Root mean square deviation; Kurt: Kurtosis; Rsk: Skewness; Min: Minimum values in the assessment; StdDev: Standard deviation measurements.

The SHAP analysis results of the model can be observed in Figure 5.

### 3.3. Image-Based Modelling

The best-performing models obtained via image-based modelling with the first approach can be seen in Table 3 below, as per the methodology described in Section 2.7. Just like for the numerical data-based models, the models were assessed on their RMSE and R^2^ values. The VGGNet-inspired architecture performed the best with a result of RMSE of 79 and R^2^ of 0.96. The difference in the accuracy of predictions is clearly due to the differences in architectures. The VGGNet-inspired architecture also performed better than the best-performing numerical data model developed with the use of AutoML by H2O. This suggests that manual retrieval of data from images may lead to the loss of information, which might have been, in fact, used by a CNN model.

The second approach, which incorporated extrapolation, also produced interesting results. The best model developed with this approach was developed with the ResNet-inspired architecture. Its results are summarised in Table 4. The R^2^ and RMSE values were calculated separately for the two formulations to see how the model differentiates between formulations and keeps the trend of Metformin concentration decreasing for both formulations. For both the IR and XR formulations, small fluctuations in predicted values are observed; however, the absorption trends and subsequent decrease in concentration are predicted.

Based on the results, it can be deduced that the model does not only differentiate between the two formulations (otherwise, it would predict the same in vivo value for the same timepoints) but that it also makes substantially accurate predictions when extrapolating.

Validation results As described in Section 2.7 Validation, the developed model, which performed well in the extrapolation experiment, was further validated with an independent data set for the immediate-release (IR) Glucophage 850 mg tablet. In vitro dissolution profiles were obtained following the procedure described in Section 2.2. The model trained on Glucophage IR 500 mg and XR 750 mg data up to 1260 min, as described in Section 2.7, was applied to generate predictions. The model reproduced the overall shape of the observed curve well, yielding an RMSE of 104.5 and an R^2^ of 0.92 on the validation set. A head-to-head comparison of the first 11 predictions for the validation data with those obtained for the training set (Glucophage IR 500 mg) is provided in Table 5. The model successfully captured the higher plasma concentrations and closely predicted C_max_ for the 850 mg tablet, although larger deviations appeared in the elimination phase. A summary of predictions for Glucophage IR 850 is presented in Supplementary Materials (S1).

## 4. Discussion

Out of all the models developed using extracted numerical data representing the problem, a model developed with h2o AutoML behaved the best, with an RMSE of 82.0 and R^2^ of 0.95. It shows that an automated machine learning system can have a better outcome in the search for optimal hyperparameters compared to the manual and systematic search. Based on both our experience and findings in the literature, automated ML systems often achieve results comparable to state-of-the-art performance by leveraging diverse models and hyperparameters. This is typically accomplished through techniques such as random search and stacked ensembles [25,30]. This automated exploration can deliver high-performing model configurations effectively, especially in complex search spaces. However, it’s important to note that the relative success of any AutoML system can be problem-dependent. Additionally, the SHAP analysis result proves the models’ use of features generated at different wavelengths and media, further proving the complexity of the relationship between in vivo absorption and in vitro multidimensional representation of the dissolution process. However, the largest limitation of this approach is the required data extraction and possible loss of information in that process. That’s why we decided to evaluate convolutional neural networks as systems capable of directly using images as the data source in prediction tasks.

In that approach, frames from SDi2 analysis were directly used in the predictive model development process with CNN algorithms. For both approaches, different CNN architectures were tested, and two separate setups were utilised: a regular 10-fold cross-validation and train-test extrapolation approach. The first approach was designed to present stacks of six SDi2 frames (resulting from three dissolution media and two acquisition wavelengths) from the in vitro dissolution time point and the corresponding metformin plasma concentration for that time point as the target value. The best model developed with this approach utilised the VGG-inspired architecture, with RMSE of 79.0 and R^2^ of 0.96. This result clearly proves that the use of the whole image can result in a good predictive model for the IVIVR problem, outperforming models developed on extracted and compressed numerical data. This observation may stem from incomplete information being provided to the model due to preceding image processing and data extraction. Convolutional Neural Networks (CNNs) offer an advantage through their ability to autonomously learn and extract relevant features directly from raw input images. This capability contrasts with traditional methods that often rely on laborious, manually extracted features. As a result, leveraging CNNs for direct image processing frequently leads to enhanced predictive accuracy in various computer vision applications, effectively replacing older techniques. Moreover, the VGG architecture was found to perform well in medical/pharmaceutical image processing [31,32].

**Figure 6:**
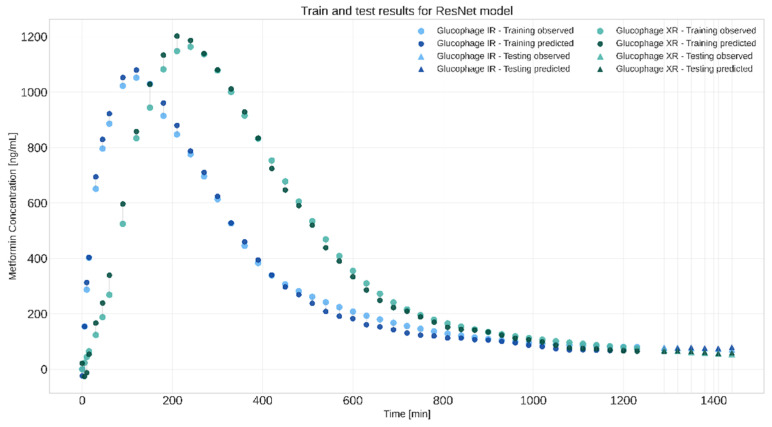
Visual representation of ResNet-inspired model in extrapolation experiment. Plot presents in vivo metformin plasma concentrations predicted by model and the observed concentrations. Data for immediate-release tablets (IR) are presented in blue, whereas data for extended-release tablets (XR)are in green. Circle symbols were used for training data points and triangles for the test data points, which predominantly represent the later time points deliberately excluded during training to assess extrapolation capability.

The second approach involved creating stacks of frames from the entire dissolution process, a time point which was represented in the form of an additional layer, and, of course, with the corresponding in vivo Metformin plasma concentration as the target value. The last few timepoints were deliberately left out to create an extrapolation scenario. The best model was obtained with the ResNet-inspired architecture yielding the best results, with an RMSE of 3.26 and R^2^ of 0.83. Train and test results in the form of in vivo metformin plasma concentration profiles are presented in Figure 6.

The model developed for the extrapolation approach was also validated against the 850 mg IR dissolution data, which the model had not seen before. The model received no explicit information about the tablet dose or formulation type (IR or XR); its predictions relied solely on the multidimensional representation of the SDi2 dissolution results. The model was able to predict the shape of the plasma concentration-time curve C_max_ value and scored well on the performance evaluation metrics, achieving an RMSE of 104.5 and an R^2^ of 0.92. These results demonstrate that the CNN model is capable of generating predictions for new, unseen data. This demonstrates the potential practical utility of the proposed multidimensional data representation and CNN-based empirical IVIVR modelling.

It is worth noting that the results depended heavily on the type of CNN architecture used, highlighting the importance of conducting research on CNN architecture types and conducting trial runs to select the architecture that works best for your data.

Previous studies explored IVIVC for Metformin formulations using conventional approaches. Mohylyuk et al. [33] investigated IVIVC for hydrophilic matrix tablets by testing the dissolution process under various mechanical stress conditions and comparing them to deconvoluted in vivo absorption profiles. Their work highlighted the challenges of matching in vitro and in vivo profiles due to high inter- and intra-individual variability and the limited availability of individual in vivo profiles. Similarly, Balan et al. [34] developed level C and level A IVIVC models for Metformin-modified-release formulations, employing both deconvolution and convolution approaches. They found that while level C models could be highly predictive, level A models were limited by Metformin’s absorption characteristics. Our study presents a proof-of-concept approach that leverages convolutional neural networks (CNNs) applied to image-based dissolution data to empirically predict in vivo plasma concentration profiles. Unlike previous work, our method utilises the full spatial and temporal information from dissolution imaging rather than relying solely on summary dissolution metrics. This approach aims to capture complex dosage form behaviours and potentially improve the prediction of in vivo performance.

## 5. Conclusions

The fact that CNN-generated models had lower R^2^ and RMSE values supports the hypothesis that the numerical data generation by manual retrieval (compression) of data from images may omit important data, which can otherwise be picked up by a CNN algorithm that processes the entire image.

The extrapolation approach model, which was validated against the unseen dataset of the 850 mg formulation, showed that it is possible to develop a model through CNN with limited data and use it to predict values for data that the algorithm did not see before. This is quite a promising result, as it shows that the model is able to make good general predictions without being overfitted to the data it was exposed to during training. While the validation results are quite promising, they could still be improved in future. The model could be improved in the future by obtaining dissolution image data for yet other formulations of Metformin (different dose, immediate or extended release), as well as corresponding in vivo data. However, with the limited amount of data available, the results presented in this study further strengthen AI’s position in the development of new pharmaceuticals, especially within dissolution testing.

The integration of DNNs into pharmaceutical sciences marks a paradigm shift in how drug dissolution and absorption are studied and understood, with far-reaching implications for the industry and patients. By offering more accurate, efficient, and data-driven approaches to analysing UV dissolution images, these technologies empower researchers to optimize formulations more rapidly, predict drug behaviour with greater confidence, and reduce the time and cost associated with bringing new drugs to market. This efficiency translates into tangible benefits, such as the ability to deliver safer, more effective medications to patients faster than ever before. Moreover, as the pharmaceutical industry increasingly embraces digital transformation, the role of deep learning is set to expand, paving the way for novel research methodologies and development strategies. From improving quality control to enabling personalized medicine, these advancements promise to unlock new opportunities that were once beyond reach, solidifying deep learning’s place as a cornerstone of pharmaceutical innovation.

## Figures and Tables

**Table 1:** Table representing the structure of data for the extrapolation approach. The R, G, B and TP stand for Red, Green, Blue, and Timepoint respectively. A single row is a single stack of images showing the entire dissolution process, with the variable TP being a 4th dimension or layer, encoding the timepoint as mentioned earlier. It can be observed that the images do not differ between rows apart from the 4th dimension being the timepoint and the corresponding output being the scaled in vivo plasma concentration. It can also be observed that the R, G, B frames differ between columns, as this portrays the dissolution process happening in between frames, while the timepoint stays the same.

Timepoint	Frame 1	…	Frame 108	In Vivo Plasma Conc. (Scaled)
0 min	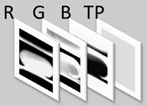	**…**	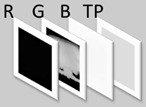	0
⋮	⋮	⋮	⋮	⋮
1440 min	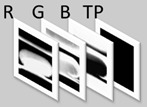	**…**	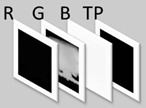	0.047

**Table 2:** Performance comparison of predictive models developed using numerical descriptors extracted from images with SurfCharJ. Models were evaluated using the 10-fold cross-validation (10-CV) method. RMSE: root mean squared error; R^2^: coefficient of determination.

	Ref.	Linear	LASSO	Ridge	ElasticNet	RF	MLP	DNN	H2O
RMSE	376	103	187	103	114	146	152	197	-
R^2^	0.0	0.92	0.75	0.92	0.91	0.85	0.84	0.73	-
After feature engineering/feature selection (H2O)									
RMSE	376	124	336	127	122	205	192	241	82
R^2^	0.0	0.89	0.2	0.89	0.89	0.7	0.74	0.59	0.95

**Table 3:** Best models obtained through image-based CNN learning algorithms. The OwnDesign1, OwnDesign2, and OwnDesign3 columns refer to architectures made by our own design with the use of a few simple convolutions, max pooling operations and batch normalization as described in the methodology Section 2.7. The Inception, ResNet, and VGG columns refer to the Inception module-based/GoogleNet-inspired architecture, Residual module-based/ResNet-inspired architecture, and the VGGNet-inspired architecture, respectively. RMSE and R^2^ are calculated in the 10-CV approach.

	OwnDesign1	OwnDesign2	OwnDesign3	Inception	ResNet	VGG
RMSE	120.0	1584.0	2090.0	727.1	231.0	79.0
R^2^	0.90	−17.0	−30.0	−2.73	0.62	0.96
No. of Iterations	200	40	150	200	850	650

**Table 4:** Table displaying observed and predicted by the best ResNet-inspired model in vivo plasma metformin concentrations for both formulations, as well as the R^2^ and RMSE values calculated for the formulations separately. IR: immediate-release tablet; XR: extended-release tablet.

Time Point [min]	Formulation	Predicted	Observed	Results
1290	IR	73.41	77.93	RMSE: 3.26 R^2^: 0.83
1320	IR	76.02	76.98	
1350	IR	78.18	75.95	
1380	IR	76.25	74.78	
1410	IR	75.42	73.41	
1440	IR	79.09	71.80	
1290	XR	65.32	68.24	
1320	XR	65.91	65.08	
1350	XR	64.53	62.11	
1380	XR	60.86	59.34	
1410	XR	57.34	56.75	
1440	XR	59.27	54.34	

**Table 5:** Summary of the validation results for the ResNet-inspired model based on in vivo plasma metformin concentrations for both formulations. IR 500 = Glucophage immediate-release tablet, 500 mg; IR 850 = Glucophage immediate-release tablet, 850 mg.

	Glucophage IR 500 (Part of Train Data set)		Glucophage IR 850 (Validation Data Set)	
Time	C_plasma_ Observed	C_plasma_ Predicted	C_plasma_ Observed	C_plasma_ Predicted
0	0	−24.66	0	−7.97
5	154.08	154.03	101.46	201.78
10	287.50	312.44	202.52	306.03
15	402.07	403.37	302.79	388.58
30	650.97	693.87	594.86	683.09
45	795.83	828.69	852.69	843.94
60	885.81	921.78	1012.76	965.14
90	1022.62	1052.07	1045.70	1085.21
120	1052.33	1079.44	1088.74	1091.99
150	1028.74	1029.44	1055.26	1026.70
180	913.82	960.40	1096.07	951.21

## Data Availability

Prepared models and the database are available on request.

## Supplementary Materials

The following supporting information can be downloaded at: https://www.mdpi.com/article/10.3390/pharmaceutics17060728/s1, S1, Summary of validation results for the ResNet-Inspired model.
