# Supplementary material for: Harnessing Real-Time UV Imaging and Convolutional Neural Networks (CNNs): Unlocking New Opportunities for Empirical In Vitro–In Vivo Relationship Modelling
Source: Pharmaceutics. 2025 May 31;17(6):728. doi: 10.3390/pharmaceutics17060728 (PMC12196273; doi:10.3390/pharmaceutics17060728)
Supplement: Supplementary file 1 [file pharmaceutics-17-00728-s001.zip › pharmaceutics-3582655-supplementary.pdf]

Suplementarny materials (S1)

Summary of validation results for the ResNet-Inspired model: Observed vs. Predicted plasma metformin concentrations for Glucophage IR 850.

Time	Observed	Predicted
0	0	-7.97
5	101.46	201.78
10	202.52	306.03
15	302.79	388.58
30	594.86	683.09
45	852.69	843.94
60	1012.77	965.14
90	1045.70	1085.22
120	1088.74	1091.99
150	1055.26	1026.70
180	1096.07	951.21
210	1047.19	864.91
240	970.89	769.00
270	884.84	689.77
300	789.04	598.92
330	688.43	501.43
360	594.16	434.03
390	515.70	368.07
420	452.15	318.40
450	400.56	276.46
480	357.96	251.88
510	321.68	224.51
540	290.12	197.30
570	261.95	183.14
600	235.84	177.61
630	210.84	156.37
660	187.27	148.12
690	165.77	137.67
720	146.97	127.78
750	131.13	121.03
780	117.64	118.61
810	105.68	110.44
840	94.47	110.65
870	83.21	104.92
